# The Complete Mitochondrial Genome of *Gynostemma pentaphyllum* Reveals a Multipartite Structure and Dynamic Evolution in Cucurbitaceae

**DOI:** 10.3390/genes17010007

**Published:** 2025-12-20

**Authors:** Ming Zhu, Yanping Xie, Caiyan Chen, Yun Han

**Affiliations:** 1 College of Life Sciences, South China Agricultural University, Guangzhou 510642, China; mzhu@scau.edu.cn (M.Z.);; 2 School of Life Sciences, Huaibei Normal University, Huaibei 253000, China

**Keywords:** *Gynostemma pentaphyllum*, mitogenome, RNA editing, phylogenomics, chloro-plast-to-mitochondrion transfer (MTPTs)

## Abstract

Background: *Gynostemma pentaphyllum* (Thunb.) Makino is an important medicinal plant within the Cucurbitaceae family. Despite its economic and pharmacological importance, genomic resources for this species remain limited. Methods: We sequenced and assembled the complete mitochondrial genome of *G. pentaphyllum*. Comparative analyses were conducted to investigate the genomic structure, gene content, RNA editing events, and intracellular gene transfer (IGT) from chloroplasts. Additionally, phylogenomic relationships, synteny, and the selective pressure on mitochondrial genes were evaluated against related species within Cucurbitaceae. Results: The ~324 kb mitogenome has a multipartite architecture of six circular-mapping molecules. It encodes the typical complement of mitochondrial protein-coding genes, tRNAs, and rRNAs found in angiosperms. Extensive C-to-U RNA editing, including events that generate functional start and stop codons, points to substantial post-transcriptional regulation. We also detected multiple chloroplast-derived fragments, including several intact genes, indicating active intracellular gene transfer. Phylogenomic analyses of conserved mitochondrial genes place *G. pentaphyllum* firmly within Cucurbitaceae, clustering it with *Thladiantha cordifolia* and *Momordica charantia*, whereas synteny comparisons reveal pronounced structural rearrangements with respect to these close relatives. While most genes evolve under strong purifying selection, *rps1*, *sdh3,* and *sdh4* show signatures of accelerated evolution; furthermore, haplotype networks based on conserved loci further corroborate the close affinity with *T. cordifolia*. Conclusions: This study provides the first high-resolution mitogenome resource for *G. pentaphyllum* and candidate mitochondrial markers for species authentication, evolutionary studies, and breeding in *Gynostemma* and related cucurbits.

## 1. Introduction

*Gynostemma pentaphyllum* (Thunb.) Makino is a perennial climbing herb in the family Cucurbitaceae, which is widely distributed across South and East Asia [1]. Commonly known as “Southern Ginseng” or “Jiao Gu Lan,” it is used in traditional Chinese medicine [2,3]. Phytochemical studies have identified dammarane-type triterpenoid saponins (gypenosides) and diverse flavonoids as major constituents associated with its anti-inflammatory, antioxidant, lipid-modulating, antiproliferative, and neuroprotective activities [4,5]. At the same time, hybridization, facultative apomixis, and polyploidy make species delimitation within *Gynostemma* notoriously difficult, especially for the widespread and morphologically variable *G. pentaphyllum* [6]. Recent chloroplast genome studies covering 21 individuals from 14 *Gynostemma* species have clarified the main phylogenetic clades, identified several hypervariable plastid regions, and also revealed highly conserved gene content, limited chloroplast polymorphism, and even a non-monophyletic pattern for *G. pentaphyllum*, indicating that plastid markers alone do not yet provide fully reliable species-level diagnostic characters for this genus [7]. Despite this medicinal and economic relevance, organellar genomic resources—particularly mitochondrial—for *G. pentaphyllum* remain incomplete, constraining both precise species authentication within *Gynostemma* and broader inferences about mitochondrial genome dynamics in Cucurbitaceae.

Beyond the nucleus, plant cells harbor two additional genetic systems within their plastids and mitochondria. These organellar genomes, which are predominantly maternally inherited in angiosperms, provide powerful perspectives on evolutionary history, tracing the lineage diversification and developing diagnostic barcodes [8]. Plant mitochondrial genomes (mitogenomes) are particularly enigmatic; while they typically retain a conserved core set of genes essential for respiration and ATP synthesis, they exhibit an astonishing degree of variation in size, structure, repeat content, and post-transcriptional modifications, such as RNA editing [9,10]. This structural plasticity is not merely an evolutionary curiosity; it has profound biological consequences. For instance, rearrangements within the mitogenome can generate novel chimeric open reading frames (ORFs) that are directly linked to important agronomic traits such as cytoplasmic male sterility (CMS), a phenomenon critical for hybrid seed production [11,12,13].

The Cucurbitaceae family represents an extreme example of this mitochondrial dynamism. Its members display an order-of-magnitude variation in mitogenome size, ranging from approximately 390 kb to over 2.9 Mb [14,15]. The immense size of some cucurbit mitogenomes, such as that of melon (*Cucumis melo*), is largely attributed to the massive incorporation of DNA from the nucleus and chloroplasts, as well as the proliferation of repetitive sequences [16]. These features often lead to complex, multipartite architectures, where the genome exists as a dynamic collection of circular-mapping molecules generated through frequent repeat-mediated recombination [17]. Furthermore, the transfer of genetic material from chloroplasts to mitochondria (MTPTs) is a pervasive evolutionary process that contributes to the structural complexity and functional landscape of plant mitogenomes [18,19]. This family therefore serves as an exceptional natural system for investigating the mechanisms that drive structural evolution and intracellular gene transfer [20,21].

Despite significant progress in sequencing other cucurbit mitogenomes, a complete reference for any species within the *Gynostemma* genus has been conspicuously absent. Consequently, fundamental aspects of its mitochondrial biology—including its architectural configuration, the role of repetitive elements in shaping its structure, the landscape of RNA editing, and the extent of MTPTs—remain entirely uncharacterized. Moreover, the limited resolution of plastid markers for species delimitation in *Gynostemma* suggests that complementary mitochondrial loci may provide additional diagnostic organellar markers for species authentication and for tracking cytoplasmic variation within the genus. To address these critical gaps, this study was designed to: (i) assemble a high-quality, curated reference mitogenome for *G. pentaphyllum* using a hybrid strategy of long- and short-read sequencing data; (ii) comprehensively characterize its genomic features, including gene content, repeat landscape, and RNA editing sites; (iii) identify and quantify MTPTs to elucidate the history of inter-organellar DNA exchange; and (iv) situate *G. pentaphyllum* within its broader evolutionary context through robust comparative, phylogenomic, and selection analyses across the Cucurbitaceae. The results provide foundational organellar resources for *Gynostemma* and offer new insights into the mechanisms driving the remarkable structural plasticity of plant mitochondrial genomes, while also delivering candidate mitochondrial loci that can be further developed as markers to support species authentication and taxonomic refinement in this medicinally important genus.

## 2. Materials and Methods

### 2.1. Plant Material, DNA/RNA Extraction, and Sequencing

Fresh leaf tissues of *G. pentaphyllum* were collected from a wild population in Jiangxi Province, China (27.52° N, 117.20° E). A voucher specimen (JGL01) was deposited at the Herbarium of South China Agricultural University for taxonomic verification. The collected leaves were immediately flash-frozen in liquid nitrogen and stored at −80 °C until use. For genomic DNA extraction, the frozen leaf tissue was ground to a fine powder in liquid nitrogen using a pre-chilled mortar and pestle. Total DNA was isolated using a modified cetyltrimethylammonium bromide (CTAB) protocol, containing 2% CTAB, 1.4 M NaCl, 100 mM Tris–HCl (pH 8.0), 20 mM EDTA, and 0.2% (*v*/*v*) β-mercaptoethanol, added immediately before use. After incubation at 65 °C for 30 min with occasional mixing, an equal volume of chloroform/isoamyl alcohol (24:1) was added, and the mixture was centrifuged to separate phases. DNA was precipitated from the aqueous phase with cold isopropanol, washed twice with 70% ethanol, air-dried, and finally dissolved in TE buffer. RNA contamination was removed by RNase A treatment. Total RNA was concurrently isolated using a BioTeke RNA extraction kit (BioTeke Corporation, Beijing, China) according to the manufacturer’s protocol. The integrity, purity, and concentration of the extracted nucleic acids were rigorously assessed via agarose gel electrophoresis and spectrophotometry. High-quality samples were then submitted to Wuhan Benagene Technology Co., Ltd. (Wuhan, China) for library preparation and sequencing on both the Illumina (short-read) and Oxford Nanopore Technologies (ONT) platforms (long-read).

### 2.2. Mitochondrial Genome Assembly and Annotation

A hybrid strategy was used to reconstruct the complete mitochondrial genome (mitogenome) of *G. pentaphyllum*. ONT long reads were assembled de novo with Flye to generate draft contigs. Illumina short reads and ONT long reads were then remapped to the draft using minimap2 with data-type-appropriate presets (ONT: map-ont; Illumina: sr) to refine the consensus. Long-read and short-read polishing followed standard practice: ONT-guided correction with racon and medaka, followed by an Illumina-guided pass with Pilon. Assembly graphs were examined in Bandage to assess repeat connectivity and to derive a non-redundant set of circular-mapping contigs [22]. Assembly correctness was corroborated by read-to-assembly alignments inspected in IGV, including junction-spanning ONT reads and locally uniform coverage around circular closures. Gene annotation was performed using a multi-tool pipeline: protein-coding genes (PCGs) were predicted with IPMGA, transfer RNA (tRNA) genes with tRNAscan-SE v2.0 [23], and ribosomal RNA (rRNA) genes via BLASTN [24] searches against a reference database. All predicted genomic features were manually inspected, curated, and corrected in Apollo v2.6.6 [25] to ensure maximum accuracy. The complete mitochondrial genome sequence used for this analysis was obtained from our previously published dataset [26].

### 2.3. Genome Feature Characterization and Variation Analysis

Potential C-to-U RNA editing sites within mitochondrial PCGs were computationally predicted using the DeepRedMT web server [27], which employs a deep learning algorithm trained on plant mitochondrial data. To identify regions of intracellular gene transfer, the complete chloroplast and mitochondrial genomes were aligned using BLASTN (E-value cutoff < 1 × 10^−5^). High-scoring homologous fragments (length ≥ 100 bp, identity ≥ 80%) were considered mitochondrial plastid DNAs and were summarized and visualized using Circos v0.69-5. To assess genetic variation, nucleotide diversity (Pi) was calculated for homologous genes. This analysis involved aligning sequences with MAFFT v7.427 [28], and Pi values were computed from the curated alignments using CPStools [29].

### 2.4. Phylogenetic Analysis

To resolve the phylogenetic position of *G. pentaphyllum* within Cucurbitaceae, we compiled a dataset of coding sequences (CDSs) from 21 selected plant mitochondrial genomes. Taxa were chosen to cover the major lineages of Cucurbitaceae while including appropriate outgroups for rooting. For each species, a common set of conserved mitochondrial PCGs present across all taxa was extracted and concatenated into a single supermatrix. Individual CDSs were aligned using MAFFT v7.427 [28] and trimmed with trimAl v1.4.rev15 (−gt 0.7) [30]. Using jModelTest v2.1.10 [31], the General Time Reversible (GTR) model was selected as the optimal substitution model. A Maximum Likelihood (ML) tree was inferred with RAxML v8.2.10 [32] under the GTRGAMMA model with 1000 bootstrap replicates to assess node support. In parallel, a Bayesian Inference (BI) tree was constructed using MrBayes v3.2.7 [33] via Markov chain Monte Carlo (MCMC) sampling for 1,000,000 generations, at every 1000 generations, discarding the first 25% of trees as burn-in.

### 2.5. Comparative Genomics and Selection Analysis

Whole-genome synteny was analyzed to assess structural conservation and rearrangements among mitogenomes. Pairwise alignments were generated using nucmer (MUMmer v4.0.0beta2) [34] with the maxmatch parameter, filtered with a delta-filter to retain the best one-to-one matches, and visualized as dot plots to highlight conserved and rearranged regions. The mitochondrial gene content evolution was investigated by identifying orthologous gene groups with OrthoFinder v2.5.4 [35], which was used to determine gene presence/absence patterns and copy number variation across species. To evaluate selective pressures on mitochondrial PCGs, we calculated pairwise non-synonymous (Ka) and synonymous (Ks) substitution rates for one-to-one orthologues. Coding sequences were aligned at the codon level with MAFFT v7.427 [28], and Ka/Ks ratios were estimated using the KaKs_Calculator v2.0 [36] under the MLWL model. A Ka/Ks ratio > 1, <1, or ≈1 was interpreted as evidence of positive selection, purifying selection, or approximately neutral evolution, respectively.

## 3. Results

### 3.1. Mitochondrial Genome Assembly and Annotation

**Figure 1:**
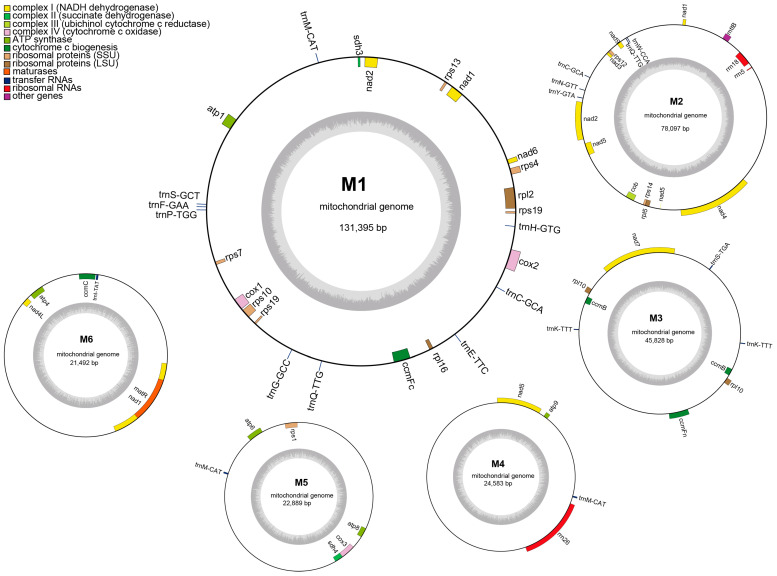
Circular representation of the mitochondrial genome of *Gynostemma pentaphyllum*. The outer ring shows genes encoded on both strands, color-coded according to functional categories, and the inner ring illustrates local GC content variation. Six circular-mapping molecules (M1–M6) are indicated.

A hybrid assembly strategy yielded the complete mitochondrial genome of *G. pentaphyllum*. The mitogenome has a total size of 324,284 bp and is organized into a complex multipartite architecture comprising six distinct circular-mapping molecules, hereafter referred to as M1–M6 for clarity (Figure 1). The mean read depths were 517× for M1, 526× for M2, 326× for M3, 456× for M4, 332× for M5 and 281× for M6, with an overall average depth of approximately 459× across the 324,284 bp mitogenome. This uniformly high coverage supports the completeness and accuracy of the mitochondrial genome assembly. The overall GC content is 45.39%. The individual molecules vary significantly in length, ranging from 21,492 bp (M6) to 131,395 bp (M1), while their GC contents are relatively stable (44.74–45.91%; Table S1). In total, the mitogenome encodes 57 genes, including 39 unique PCGs, 15 tRNA genes, and three rRNA genes (Table S2). Gene duplication was detected for three PCGs (*ccmB*, *rpl10*, and *rps19*), and several tRNA genes occurred in multiple copies, likely associated with repetitive regions: *trnC-GCA* (two copies), *trnK-TTT* (two copies), *trnQ-TTG* (two copies), and *trnM-CAT* (three copies). The lengths of the annotated genes fall within the typical range for plant mitogenomes: PCGs span 225–1947 bp, tRNAs 71–87 bp, and rRNAs 118–3392 bp.

### 3.2. Prediction of RNA Editing

**Figure 2:**
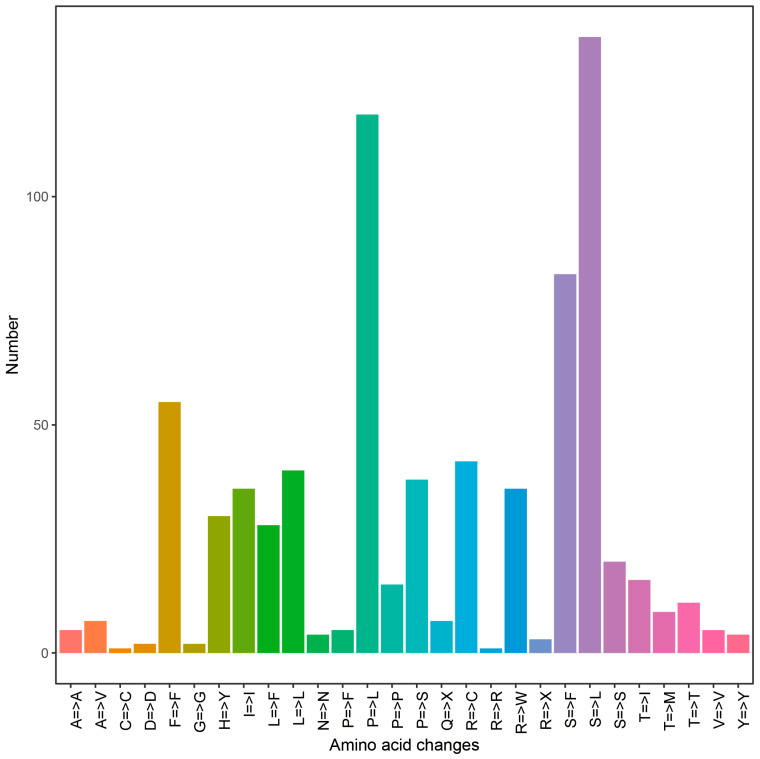
Number of RNA editing sites by type of amino acid change across 39 mitochondrial PCGs.

We predicted a total of 850 C-to-U RNA editing sites across the 39 mitochondrial PCGs, indicating extensive post-transcriptional modification (Figure 2; Table S3). The editing frequency varied markedly among genes and was generally highest in those related to cytochrome c maturation and respiration (for example, *ccmB*, *nad4*, and *mttB*), whereas ribosomal protein genes such as *rpl2* and *rps14* contained very few sites. Of the 758 editing sites located within coding regions, the majority (72.56%; 550 sites) were nonsynonymous, leading to amino acid substitutions, while 27.44% (208 sites) were synonymous. Most substitutions increased protein hydrophobicity and the most frequent amino acid change was serine to leucine (S → L), followed by proline to leucine (P → L) (Figure 2). Functionally, RNA editing was predicted to restore canonical start codons (via ACG-to-AUG conversion) in eight genes, including key respiratory components such as *cox1* and *nad1,* while introducing premature stop codons in another eight genes (for example, *atp6* and *matR*), suggesting a potential role in fine-tuning mitochondrial protein expression (Table S4).

### 3.3. Nucleotide Diversity Varies Across Mitochondrial Genes

**Figure 3:**
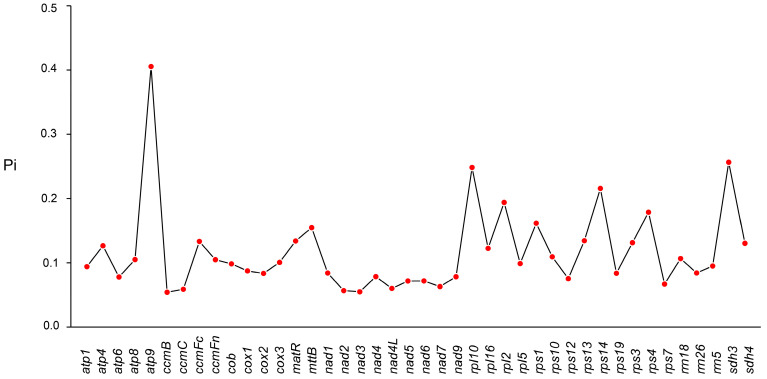
Nucleotide variability (Pi) analysis of mitochondrial genomes among *Benincasa hispida*, *Citrullus lanatus*, *Cucurbita pepo*, *Gynostemma pentaphyllum*, *Lagenaria siceraria*, *Momordica charantia*, and *Thladiantha cordifolia*.

We estimated pairwise nucleotide divergence (π; mean pairwise differences per site) for 42 mitochondrial PCGs across *G. pentaphyllum* and six other cucurbits, *Cucurbita pepo*, *Benincasa hispida*, *Citrullus lanatus*, *Lagenaria siceraria*, *Momordica charantia*, and *Thladiantha cordifolia*. Across all alignments, 1182 variable sites were identified (Table S5). The gene-wise π varied markedly, ranging from 0.00552 to 0.04136 (Figure 3). *atp9* exhibited the highest divergence (π = 0.04136), whereas *ccmB* was the most conserved (π = 0.00552). These results indicate heterogeneous divergence among mitochondrial genes across Cucurbitaceae and highlight comparatively fast- versus slow-evolving loci that may be useful for downstream comparative and phylogenomic analyses.

### 3.4. Intracellular Gene Transfer from the Chloroplast

**Figure 4:**
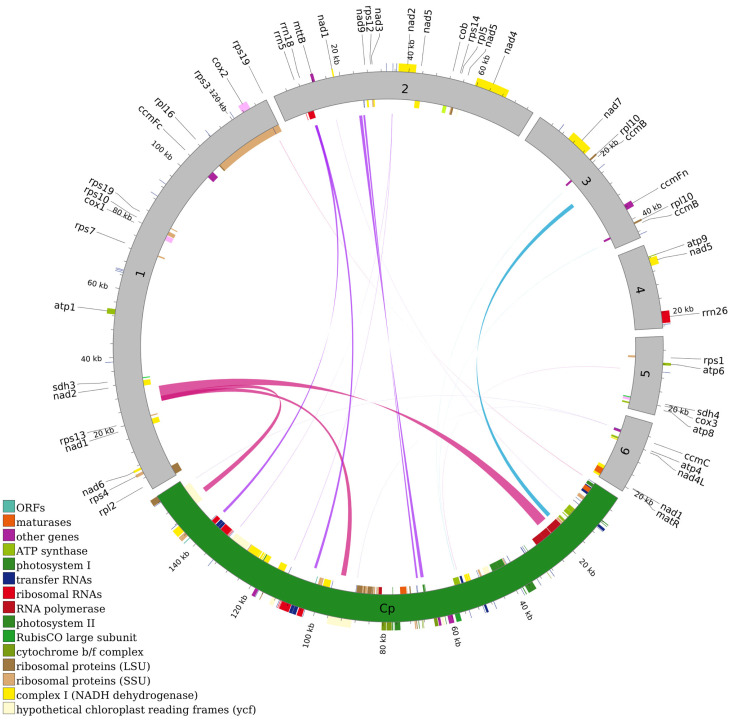
Homologous sequences between chloroplast and mitochondrial gene sequences in *Gyno stemma pentaphyllum*. The gray circular segment represents the mitochondrial sequences, while the green circular segment represents the chloroplast sequences. Colored lines connecting the two circles indicate homologous fragments, with different colors corresponding to distinct genes or genomic regions.

Analysis of inter-organellar DNA transfer identified 17 homologous fragments of chloroplast origin within the *G. pentaphyllum* mitogenome (Figure 4). These mitochondrial plastid DNAs range in size from 37 to 3550 bp, with a cumulative length of 12,371 bp, constituting 3.81% of the total mitogenome (Table S6). Among these transferred segments, ten represent complete and apparently intact chloroplast genes: four tRNA genes (*trnH-GUG*, *trnW-CCA*, *trnN-GUU*, *trnM-CAU*) and six photosynthesis-related PCGs (*psbJ*, *psbL*, *psbF*, *psbE*, *petL*, *petG*). Several MTPTs retain very high sequence identity to their plastid counterparts (>99%), suggesting relatively recent transfer events, whereas others are more diverged and likely represent older insertions.

### 3.5. Phylogenetic Analysis

**Figure 5:**
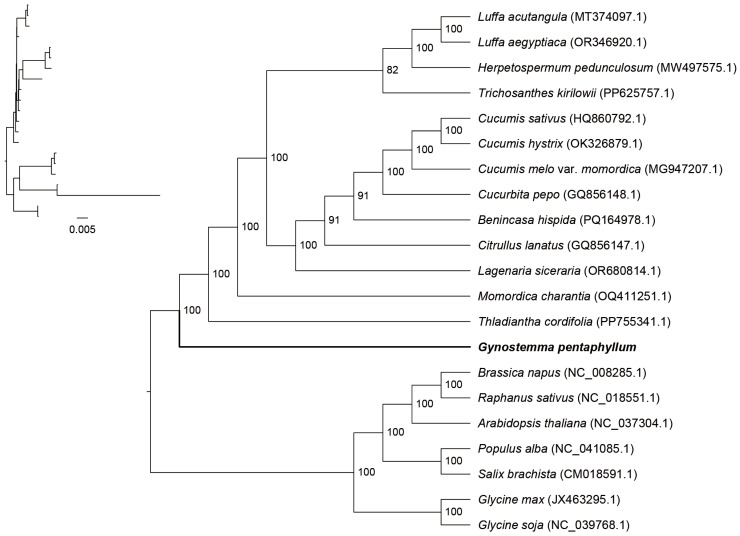
Bayesian phylogenetic tree constructed from the CDSs of 21 selected plant mitochondrial genomes. Bootstrap support values are shown at the corresponding nodes.

To resolve the evolutionary position of *G. pentaphyllum*, a phylogenetic tree was constructed using the concatenated sequences of 36 conserved PCGs from 21 species. The Bayesian Inference (BI) (Figure 5) and Maximum Likelihood (ML) (Figure S1) analyses yielded highly congruent topologies. *Gynostemma pentaphyllum* was placed confidently within a well-supported Cucurbitaceae clade (Bootstrap Support [BS] = 100%). Within this family, *G. pentaphyllum* formed a distinct sister group with *T. cordifolia* and *M. charantia*. Other major subclades within Cucurbitaceae were also resolved with high support, confirming the robustness of the phylogenetic placement.

### 3.6. Comparative Genomics Reveals Extensive Rearrangement and Dynamic Gene Content

**Figure 6:**
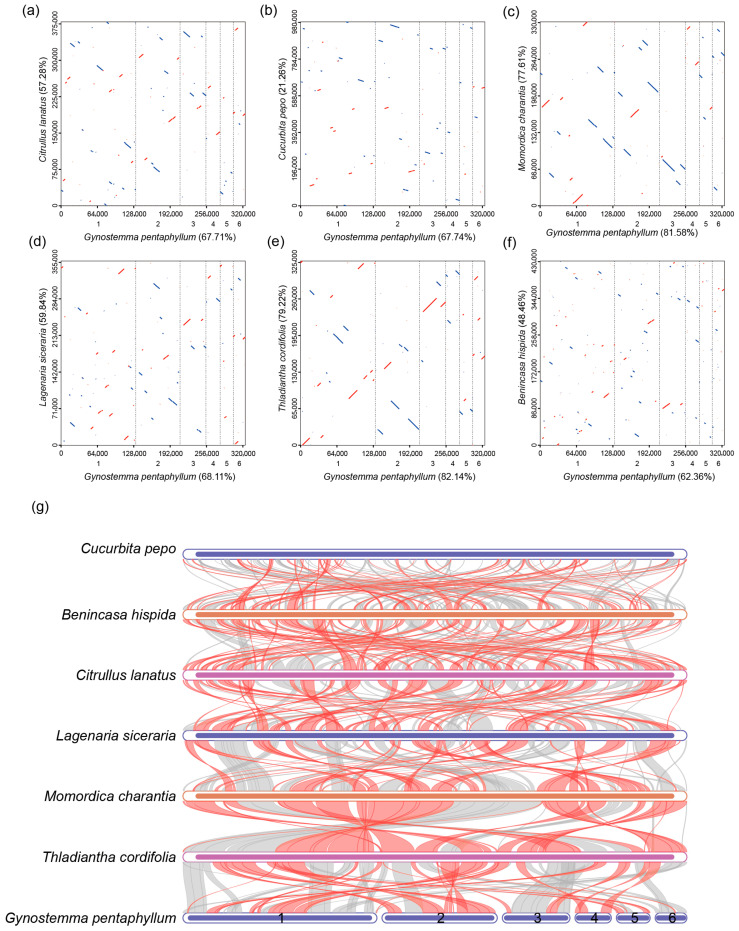
Dot plot analysis (**a**–**f**) and collinearity analysis (**g**) of mitochondrial genomes among *Gyno stemma pentaphyllum*, *Citrullus lanatus*, *Cucurbita pepo*, *Momordica charantia*, *Tinospora cordifolia*, *Lagenaria siceraria*, and *Benincasa hispida*. In each panel, the horizontal axis represents the assembled mitochondrial sequence of *G. pentaphyllum*, while the vertical axis denotes those of other species. The values in parentheses indicate the proportion of homologous sequences relative to the total mitochondrial genome length. Red lines in the dot plots represent forward alignments, whereas blue lines indicate reverse-complementary alignments. In the collinearity plot, red arcs highlight inverted regions, and gray arcs denote homologous regions with high sequence similarity. Six circular-mapping conformations (M1–M6) of the *G. pentaphyllum* mitogenome are shown.

Whole-genome synteny analysis revealed a low degree of structural conservation between the *G. pentaphyllum* mitogenome and those of other *Cucurbitaceae* species, such as *C. lanatus* and *C. pepo* (Figure 6). Although more collinear blocks were observed with its closest relatives *M. charantia* and *T. cordifolia*, these regions were highly fragmented and rearranged, indicating a history of extensive structural evolution that has reshaped the genome architecture. Comparative analysis of gene content across seven cucurbit mitogenomes identified a core set of 37 shared PCGs but also highlighted lineage-specific gene losses and gains (Figure S2). Notably, *rps14*, which has been lost in *C. lanatus*, *C. pepo*, and *B. hispida*, is retained in the lineage containing *G. pentaphyllum*, *M. charantia*, and *T. cordifolia*. Furthermore, these three species harbor multiple copies of *rps10*, a feature not observed in the other species examined, suggesting a distinct evolutionary trajectory for this ribosomal protein gene in this clade.

### 3.7. Selective Pressure Analysis

**Figure 7:**
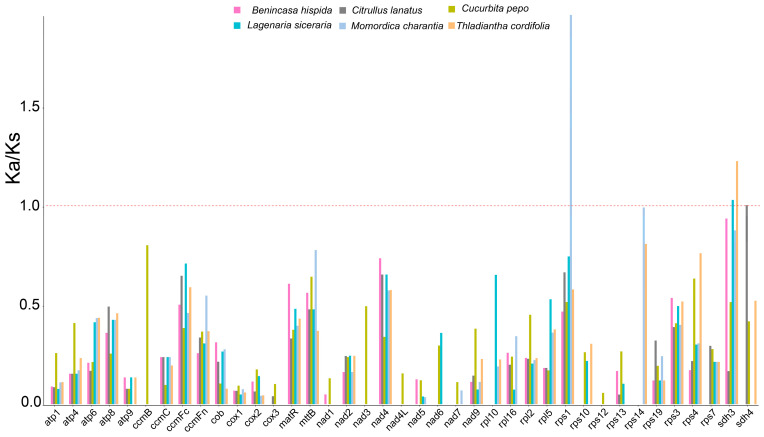
Ka/Ks ratios of protein-coding genes in 39 PCGs of *Gynostemma pentaphyllum* vs. *Benincasa hispida*, *Citrullus lanatus*, *Cucurbita pepo*, *Lagenaria siceraria*, *Momordica charantia*, *Tinospora cordifolia*, respectively. Colors denote comparison species. The dashed line at Ka/Ks = 1 indicates neutrality; values < 1 suggest purifying selection; values >1 indicate accelerated evolution in the pairwise test.

To assess the evolutionary forces acting on mitochondrial genes, we calculated the ratio of nonsynonymous to synonymous substitution rates (Ka/Ks) for one-to-one orthologous PCGs. Among 39 analyzed genes, the vast majority (35 genes) had Ka/Ks ratios consistently below 1, indicating strong purifying selection (Figure 7). By contrast, three genes exhibited Ka/Ks ratios > 1 in specific pairwise comparisons, suggesting episodes of positive selection: *rps1* (versus *M. charantia*), *sdh3* (versus *L. siceraria* and *T. cordifolia*), and *sdh4* (versus *C. lanatus*). Several core respiratory genes, including *cox3* and multiple *nad* subunits, had Ka/Ks ratios close to zero, reflecting extreme functional constraint.

### 3.8. Haplotype Network Analysis

**Figure 8:**
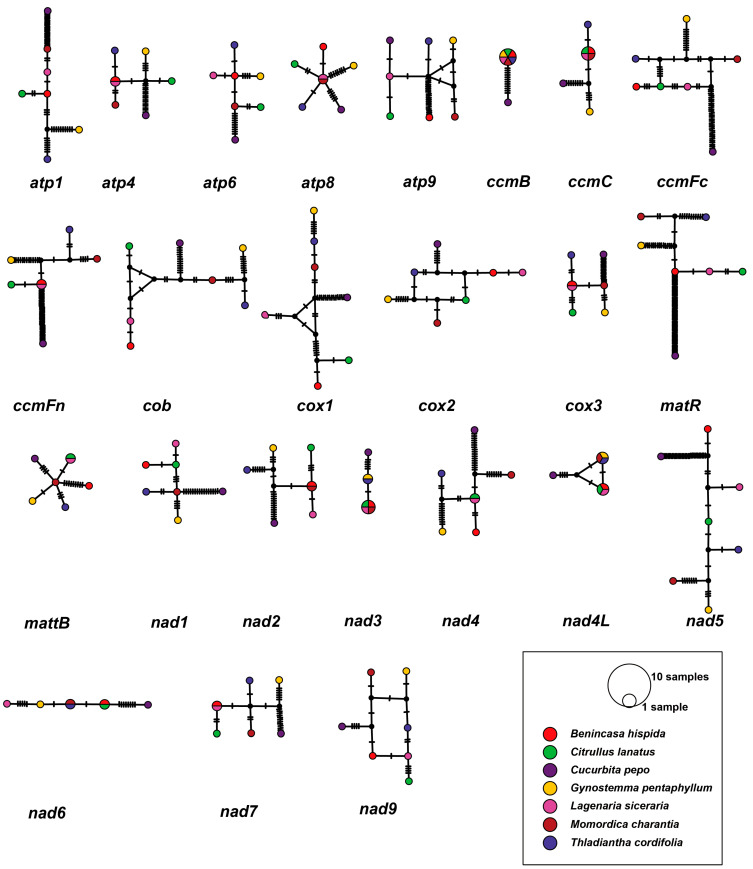
Haplotype network of 24 genes among seven *Cucurbitaceae* species. Each circle represents a distinct haplotype, with circle size proportional to the number of individuals sharing that haplotype. Colors indicate different species. Connecting lines represent mutational steps between haplotypes, and small black dots indicate inferred intermediate haplotypes not observed in the sampled sequences.

Haplotype network analysis of 24 mitochondrial genes revealed heterogeneous patterns of sequence variation patterns among the seven cucurbit species examined. Several genes, including *atp1*, *atp6*, and *cox1*, showed relatively high haplotype diversity, with each species possessing a distinct haplotype, whereas *ccmB* was highly conserved, with only two haplotypes detected across all species (Figure 8). Overall, the networks were consistent with a close similarity between *G. pentaphyllum* and *T. cordifolia* for the loci analyzed: three loci (*ccmB*, *nad3*, and *nad4L*) shared identical haplotypes between the two species, and other genes such as *cox1* and *nad1* differed by only a single mutational step. In contrast, genes such as *atp6* and *mttB* were separated by two mutational steps, indicating relatively greater divergence at these loci. The analysis also showed that *B. hispida* and *L. siceraria* share four gene haplotypes, suggesting closer similarity between them than between *L. siceraria* and *C. lanatus* for the same set of loci.

## 4. Discussion

This study presents the first complete and curated mitochondrial genome for the genus *Gynostemma*, revealing a complex multipartite architecture and offering significant insights into the evolutionary dynamics of Cucurbitaceae mitogenomes. Our findings not only resolve the structural ambiguity of the *G. pentaphyllum* mitogenome but also highlight the intricate interplay of structural rearrangement, post-transcriptional regulation, and inter-organellar gene transfer that characterizes this remarkable plant family.

### 4.1. A Multipartite Architecture Consistent with Cucurbitaceae Dynamism

The most striking feature of the *G. pentaphyllum* mitogenome is its organization into six circular-mapping molecules, a pattern consistent with the complex multipartite architectures frequently observed in Cucurbitaceae [37,38]. At 324,284 bp, it resides at the smaller end of the family’s wide size spectrum, which can exceed 2.7 Mb [16]. This relatively compact size, coupled with a stable GC content (45.39%) within the conserved range for cucurbits, suggests that although *G. pentaphyllum* partakes in the characteristic structural dynamism of the family, it has avoided the massive genomic expansions driven by large-scale nuclear DNA integration seen in relatives such as melon [39,40]. The multipartite structure is likely maintained by recombination across repetitive elements, underscoring the fluidity of plant mitochondrial genomes and challenging the outdated concept of a single “master circle” chromosome [41]. Notably, recent work using high-accuracy long reads (PacBio HiFi) has shown that improved read accuracy and repeat resolution can substantially strengthen mitogenome reconstruction and the interpretation of alternative configurations, providing a clear path for future validation of cucurbit mitochondrial structural dynamics [42]. Although we did not explicitly quantify isoform stoichiometry, the presence of multiple circular-mapping molecules is consistent with substoichiometric shifting, whereby the relative abundance of alternative mitochondrial configurations changes across tissues or developmental stages, with potential consequences for gene dosage and expression.

### 4.2. Extensive RNA Editing as a Key Post-Transcriptional Regulatory Layer

Our analysis identified 850 C-to-U RNA editing sites, a number comparable to or exceeding that of other sequenced cucurbits like watermelon [43,44]. This extensive editing landscape underscores the importance of RNA editing as a post-transcriptional regulatory layer in *G. pentaphyllum*. The functional consequences are substantial: the majority (72.56%) of edits were nonsynonymous, altering the encoded protein sequence, and these changes predominantly increased the protein hydrophobicity, which could enhance the stability and membrane integration of mitochondrial proteins. RNA editing was also predicted to restore canonical start codons in eight key respiratory genes (e.g., *cox1*, *nad1*) and to introduce premature stop codons in eight others (e.g., *atp6*, *matR*). The former is essential for proper translation, whereas the latter may provide a mechanism to modulate protein abundance. The high density of editing in cytochrome c maturation genes (*ccm* family) is particularly noteworthy, as their post-transcriptional refinement likely represents an evolutionary strategy to ensure the fidelity of the electron transport chain, compensating for a structurally dynamic genome [45,46].

### 4.3. Phylogenetic Placement and the Paradox of Structural Divergence

Our phylogenomic analysis robustly places *G. pentaphyllum* within Cucurbitaceae, forming a well-supported clade with *T*. *cordifolia* and *M*. *charantia*. This placement is strongly corroborated by our haplotype network analysis, which revealed shared identical haplotypes for three genes (*ccmB*, *nad3*, *nad4L*) between *G. pentaphyllum* and *T. cordifolia*, signifying either a very recent shared ancestry or intense functional constraint on these loci. However, this close phylogenetic relationship stands in stark contrast to the divergent structural organization of their mitogenomes. The extensive rearrangements revealed by synteny analysis, even with its closest relatives, exemplify a classic paradox in plant mitogenome evolution: a highly conserved gene content coexisting with a fluid and rapidly reorganizing genome architecture [44,47,48]. Recent syntheses further emphasize that no single mechanism fully explains this paradox, and highlight roles for homologous recombination-associated repair and homologous template availability (e.g., copy number) in shaping mutation patterns and substitution-rate variation in plant organellar DNA [49]. This structural divergence, plausibly driven by double-strand break repair and repeat-mediated recombination, underscores that gene order is an unreliable phylogenetic marker in Cucurbitaceae and highlights the necessity of sequence-based methods for inferring relationships in this family [50,51].

### 4.4. Signatures of Selection and Adaptive Evolution

The evolutionary forces shaping the *G. pentaphyllum* mitogenome are not uniform. Most genes are under strong purifying selection (Ka/Ks < 1), reflecting their essential roles in cellular respiration. By contrast, we detected signals consistent with positive selection (Ka/Ks > 1) in three genes: *rps1*, *sdh3*, and *sdh4*. These are not random genes: *sdh3* and *sdh4* encode subunits of Complex II, which links the tricarboxylic acid cycle to the electron transport chain, whereas *rps1* is a ribosomal protein crucial for mitochondrial translation [52,53]. The accelerated evolution of these genes may reflect lineage-specific adaptations to the particular metabolic demands or environmental conditions experienced by *G. pentaphyllum*, making them prime candidates for future functional and physiological studies. Heterogeneity in evolutionary rates is also evident in nucleotide diversity: genes such as *atp9* are highly divergent across cucurbits, whereas *ccmB* is extremely conserved, highlighting a spectrum of loci that may be suited to different applications, from population-level analyses to species-level barcoding [54].

### 4.5. Intracellular Gene Transfer and Implications for Future Research

The *G. pentaphyllum* mitogenome contains 17 distinct fragments of chloroplast origin, totaling 12,371 bp (3.81%) and including ten apparently intact chloroplast genes. While their functional expression in the mitochondrion is unlikely, these MTPTs serve as a molecular fossil record of the ongoing “dialogue” between organelles [55,56]. Several MTPTs retain very high sequence identity to their plastid counterparts (>99% over several hundred base pairs), consistent with relatively recent transfer events, whereas shorter and more diverged fragments likely represent older insertions that have accumulated mutations over longer evolutionary timescales. The overall proportion of MTPTs is comparable to that observed in other cucurbits, reinforcing the view that organelle-to-organelle DNA transfer is a continuous and lineage-specific process. Importantly, PacBio HiFi–based mitogenome studies have also highlighted that high-accuracy long reads can improve the delineation of intracellular gene transfer fragments and their genomic context, suggesting that future HiFi-backed assemblies and read-supported validation could further refine MTPT boundaries and clarify their structural associations with repeats/recombination hotspots [42]. Together with the repeat catalog, these MTPT-rich regions provide valuable targets for future work on substoichiometric shifting and recombination hotspots; for example, by tracking isoform stoichiometry or expression under different developmental stages or environmental conditions.

## 5. Conclusions

In this study, we assembled and annotated the first complete mitochondrial genome for the genus *Gynostemma*, providing a high-resolution view of the complex genomic landscape of *G. pentaphyllum*. The mitogenome displays a multipartite architecture composed of six circular-mapping molecules, a structural hallmark consistent with the It is actively shaped by extensive post-transcriptional modification, as reflected by a rich landscape of C-to-U RNA editing, and by ongoing intracellular gene transfer, as evidenced by multiple chloroplast-derived fragments. Phylogenomic and haplotype network analyses robustly place *G. pentaphyllum* as a close relative of *T. cordifolia*, yet detailed synteny comparisons reveal extensive structural rearrangements even among these closely related species, underscoring the rapid and divergent architectural evolution characteristic of this family. While strong purifying selection has conserved the majority of the core mitochondrial genes, compelling signals of positive selection in key respiratory and ribosomal protein genes (*rps1*, *sdh3*, and *sdh4*) point to lineage-specific adaptive evolution that warrants further functional investigation. Beyond clarifying the mitochondrial architecture and evolutionary position of this medicinal plant, our work delivers a set of candidate mitochondrial loci and structural features—including fast- and slow-evolving genes, MTPT-rich regions, and positively selected genes—that can be exploited in future research. These resources offer new opportunities for developing organellar markers for species authentication and taxonomic refinement within *Gynostemma*, for exploring cytoplasmic diversity in cucurbits, and for integrating mitochondrial information into molecular breeding programs. More broadly, the *G. pentaphyllum* mitogenome adds to a growing body of evidence that plant mitochondrial genomes represent highly dynamic yet functionally constrained systems, shaped by the interplay of structural rearrangement, inter-organellar gene transfer and adaptive evolution.

## Data Availability

The mitochondrial genome assemblies and related datasets generated in this study are available in Figshare at https://doi.org/10.6084/m9.figshare.30444230.v3 (accessed on 25 October 2025). The data used in this study are already entirely in the NCBI database (https://www.ncbi.nlm.nih.gov (accessed on 25 March 2025)).

## Supplementary Materials

The following supporting information can be downloaded at: https://www.mdpi.com/article/10.3390/genes17010007/s1, Figure S1. Maximum-likelihood phylogenetic tree based on 21 selected plant mitochondrial genomes, including *Gynostemma pentaphyllum* and related species. Bootstrap support values are shown at the corresponding nodes. Figure S2. Comparison of mitochondrial genes among seven Cucurbitaceae species. White rectangles represent conserved genes with unchanged, red rectangles denote gene duplication events and dark gray rectangles indicate gene copy losses. Table S1. Six distinct contigs identified in the mitochondrial genome of *Gynostemma pentaphyllum.* Table S2. Functional classifications and physical locations of genes in the mitochondrial genome of *Gynostemma pentaphyllum* mitochondrial genome. Table S3. Number and locations of RNA editing sites identified in all PCGs. Table S4. Prediction of RNA editing sites in the mitochondrial genome of *Gynostemma pentaphyllum.* Table S5. Genetic diversity of PCGs in the mitochondrial genome of *Gynostemma pentaphyllum.* Table S6. Homologous fragments identified between the mitochondrial and chloroplast genomes of *Gynostemma pentaphyllum.* Table S7. Syntenic blocks identified among the mitochondrial genomes of *Gynostemma pentaphyllum* and six related species.
