# Supplementary figures and images for: The Complete Mitochondrial Genome of Gynostemma pentaphyllum Reveals a Multipartite Structure and Dynamic Evolution in Cucurbitaceae
Source: Genes (Basel). 2025 Dec 20;17(1):7. doi: 10.3390/genes17010007 (PMC12840931; doi:10.3390/genes17010007)

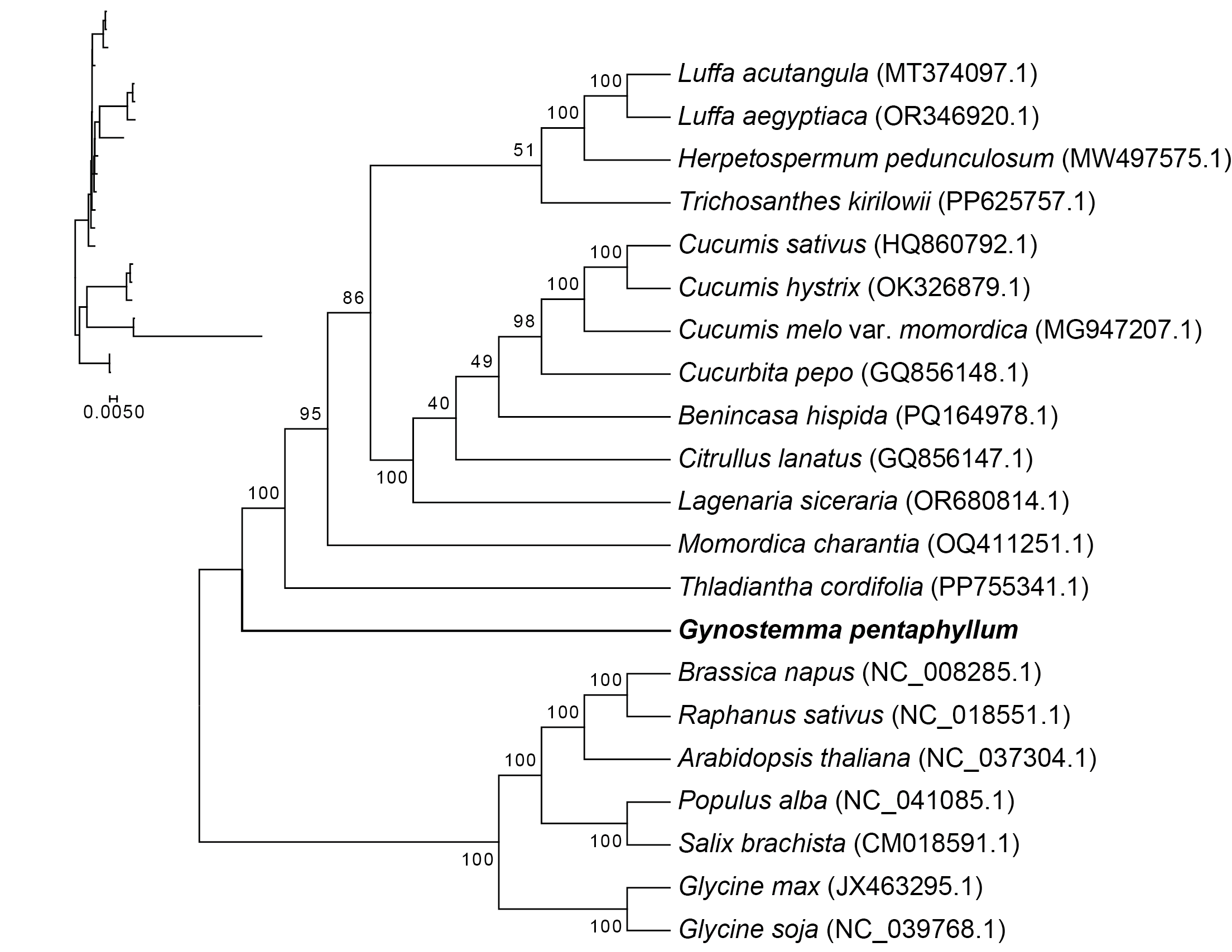

Supplement: Supplementary file 1 [file genes-17-00007-s001.zip › Figure S1.tif]

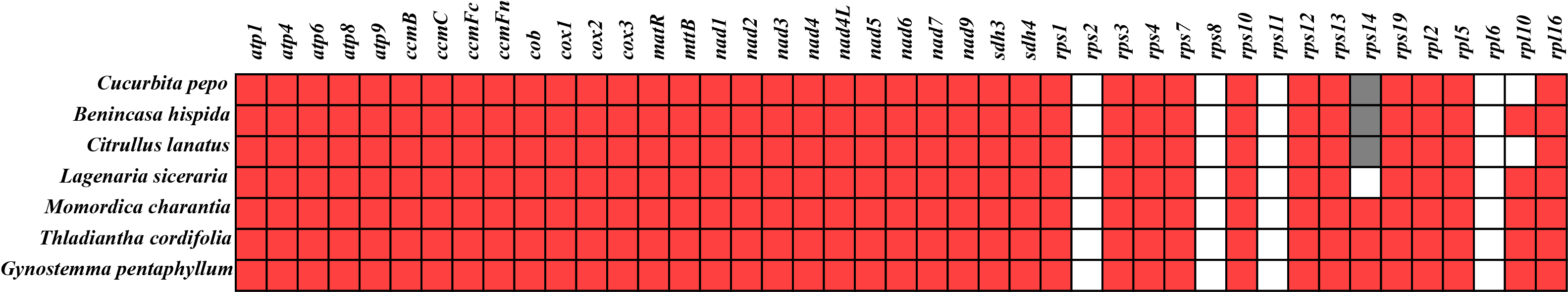

Supplement: Supplementary file 1 [file genes-17-00007-s001.zip › Figure S2.tif]
